# Salivary-Based Cell-Free Mitochondrial DNA Level Is an Independent Prognostic Biomarker for Patients with Head and Neck Squamous Cell Carcinoma

**DOI:** 10.3390/jpm13020301

**Published:** 2023-02-08

**Authors:** Lana Sayal, Omar Hamadah, Aroub AlMasri, Majdy Idrees, Issam Kassem, Wafa Habbal, Buthainah Alsalamah, Omar Kujan

**Affiliations:** 1 Department of Oral Medicine, The Faculty of Dental Medicine, Damascus University, Damascus P.O. Box 30621, Syria; 2 Biomedical Department, National Commission for Biotechnology, Damascus P.O. Box 31902, Syria; 3 UWA Dental School, The University of Western Australia, Nedlands, WA 6009, Australia; 4 Faculty of Science, Damascus University, Damascus P.O. Box 30621, Syria; 5 Clinical Laboratories Department, Al-Assad Hospital, Damascus P.O. Box 10769, Syria; 6 Department of Molecular Biology, National Commission for Biotechnology, Damascus P.O. Box 31902, Syria

**Keywords:** head and neck squamous cell carcinoma, cell-free nuclear DNA, cell-free mitochondrial DNA, saliva-based liquid biopsy, overall survival

## Abstract

Changes in the copy numbers of cell-free nuclear DNA (cf-nDNA) and cell-free mitochondrial DNA (cf-mtDNA) have shown promising diagnostic utilities among patients with head and neck squamous cell carcinoma (HNSCC). Considering the absence of objective prognostic tools for HNSCC surveillance, this study aimed to assess the utility of saliva-based cf-nDNA and cf-mtDNA in predicting the overall survival of patients with HNSCC. The study included ninety-four patients with a confirmed HNSCC diagnosis with a mean follow-up time of 32.04 months (±19.1). A saliva-based liquid biopsy was collected from each patient. A multiplex quantitative PCR was used to determine the absolute number of cf-nDNA and cf-mtDNA. The Kaplan–Meier estimator and Cox proportional hazards regression models were used to assess overall survival. The absolute copy numbers of cf-nDNA and cf-mtDNA were statistically significantly higher among the deceased patients than among the censored ones (*p* < 0.05). Individuals with elevated levels of cf-nDNA or cf-mtDNA were associated with a significantly poorer overall survival (*p* ≤ 0.05). A univariate analysis showed that only the absolute copy number of cf-mtDNA was the sole predictor of overall survival. However, the multivariate analysis showed that all the absolute copy numbers of cf-nDNA, the absolute copy numbers of cf-mtDNA, and the stage of HNSCC were predictors of overall survival. Our study confirms that saliva is a reliable and non-invasive source of data that can be used to predict the overall survival of patients with HNSCC, where cf-mtDNA levels act as the sole predictor.

## 1. Introduction

Head and neck squamous cell carcinomas (HNSCCs) account for more than 90% of malignancies involving the head and neck region [1]. Whilst the incidence of HNSCC is anticipated to increase by 40% in 2040 (which is almost 1 million new cases annually) [2], it has aggressive clinical behaviour with unpredictable survival rates and considerable functional and aesthetic compromises [1,3]. This explains why HNSCC survivors are almost twice as likely to commit suicide than survivors of the top 20 cancers in the United States [4].

Predicting the outcomes of HNSCCs is crucial for optimal therapeutic and rehabilitation planning [5]. The current approach to predicting the survival rates of patients with HNSCC is primarily based on several factors, while the most critical one is the tumour–node–metastasis (TNM) system [6,7]. The TNM system includes guidelines to interpret its elements, such as determining the depth of tumour invasion and extranodal extension [7]. However, it has been reported that the prognostic impact of the TNM system is subsite-dependent, and it is frequently difficult to apply the system across diverse presentations of squamous cell carcinoma (SCC) [6,8]. This was attributed to many factors, such as site-related genetic pathways, diverse head and neck region anatomy, and improper specimen location and orientation [6,8,9,10]. Thus, misjudging patients’ estimated survival is not uncommon due to interobserver and intraobserver variabilities among clinicians and pathologists [6].

Recent research in molecular pathology has identified thousands of biomarkers related to HNSCC. While promising outcomes have been reported when employing molecular biomarkers to detect and diagnose oral cancers and potentially malignant oral disorders [11,12], the reliability of prognostic biomarkers in HNSCC is uncertain due to the unsatisfactory quality levels of relevant studies and the heterogeneity of reporting [13]. Liquid biopsies, however, are reliable sources of certain body-fluid-based biomarkers, such as cell-free nuclear DNA (cf-nDNA) and cell-free mitochondrial DNA (cf-mtDNA) [14]. Therefore, liquid biopsies have received considerable attention for their utility as a minimally invasive tool for cancer detection and surveillance [15]. Among various body fluids that can be collected through liquid biopsies, saliva is superior over others in the head and neck region for being non-invasive and preferentially enriched with tumour DNA [15,16].

Cf-nDNA, as short fragments of DNA released into body fluids, has been established as a sensitive and reliable biomarker that reflects underlying cellular changes [17]. Although the biological properties of cf-nDNA are yet to be fully illustrated, cf-nDNA has been considered a viable marker of health, as it provides real-time snapshots of genetic aberrations [17,18]. In previous studies in patients with cancer, previous studies reported a significant increase in the released levels of cf-nDNA among patients with solid cancers, highlighting potential applications for the early detection of human malignancies [19]. Likewise, poor patient outcomes have been correlated with high concentrations of cf-nDNA among patients with various malignancies, such as prostate cancer and breast cancer [20,21].

Cf-mtDNA significantly contributes to released DNA and exhibits a higher mutation rate than cf-nDNA because it lacks an efficient DNA repair system and protective histones [22]. Evolving evidence introduced cf-mtDNA as a more efficient marker than cf-nDNA because of its simpler organisation and higher concentration in body fluids [23]. Previous findings reported a significant increase in the cf-mtDNA levels among patients with HNSCC compared to others [19,24]. Furthermore, it has been reported that patients with prostate cancer with elevated levels of plasma cf-mtDNA have lower survival rates than those with low plasma levels [25].

This project represents a part of our ongoing research to assess the utility of using saliva-based liquid biopsies for HNSCC diagnoses and surveillance [19]. The literature includes limited studies that assessed the prognostic value of cf-nDNA and cf-mtDNA among patients with HNSCC. Thus, the major aims of this study were to quantitively assess the levels of saliva-based cf-nDNA and cf-mtDNA among a longitudinally followed cohort of patients with HNSCC and to determine the prognostic utility of these biomarkers.

## 2. Materials and Methods

### 2.1. Study Design and Data Sets

This quantitative prospective cohort study was granted ethics approval from the Damascus University Human Ethics Committee (1065–2019) and was conducted in compliance with the principles of the Declaration of Helsinki and per the Reporting Recommendation for Tumor Marker Prognostic Studies (REMARK) [26]. Cases were selected from those accumulated by us between January 2019 and December 2020 for ongoing projects to assess the utility of saliva-based liquid biopsies in managing patients with HNSCC [19]. The cut-off day for the follow-up program was the end of January 2023. The overall survival of each patient was calculated from the date of diagnosis till (i) the time of death for deceased patients or (ii) the end of the project follow-up period for censored ones.

Cases were considered eligible for inclusion if they met the following criteria: (i) age of more than 18 years and signed informed consent, (ii) a confirmed microscopic diagnosis of either oral SCC or laryngeal SCC, (iii) no history of other malignancies, (iv) no history of receiving chemotherapy or radiotherapy, (v) no history of immunotherapy of immunodeficiency disorders, and (vi) the availability of complete follow-up data by the study cut-off day. The exclusion criteria were those with chronic systematic conditions, including pregnancy, and those who voluntarily decided to withdraw from the study.

Two senior pathologists blindly reviewed microscopic slides to confirm the diagnosis based on the World Health Organization (WHO) classifications [1]. The stage of HNSCC was determined as per the Eighth Edition American Joint Committee on Cancer staging Manual (TNM system) [7].

### 2.2. Saliva Collection

Saliva was collected once from each participant immediately before undergoing a surgical biopsy as described previously [27]. Briefly, the participants were asked to rinse their mouths with sterile water and to refrain from eating or drinking for at least 2 h prior to sample collection. A special saliva collection kit containing DNA-stabilising agents (PAXgene Saliva Collector, Ref. No 769040, Qiagen, Hilden, Germany) was used to collect unstimulated whole saliva according to the manufacturer’s instructions.

### 2.3. DNA Extraction and Quantitative PCR Amplification

The saliva samples were stored for up to 24 h at 4 °C after collection until being centrifuged twice at 1600 and 2600 rounds/minutes for 10 min each. The saliva supernatant was then aspirated and stored at −80 °C until further analyses. DNA was extracted from the supernatants using a DNeasy Blood and Tissue Kit (Ref. No 69504, Qiagen) according to its manufacturer’s instructions. The purified saliva DNA was quantified using a Qubit dsDNA HS and BR Assay Kit (Ref. No Q32850, Invitrogen, Waltham, MA, USA) with a Qubit 4.0 Fluorometer (Ref. No Q33238, Invitrogen).

Multiplex quantitative PCR assays were carried out in triplicate to quantify cf-nDNA and cf-mtDNA using two human-specific primers, human beta-2-microglobulin (M17987) and human mitochondrial (NC-012920), as shown in Table 1. A reaction mixture of 20 μL for each assay contained 11.5 μL of Luna Universal Probe qPCR Master Mix (Ref. No M3004X, New England BioLabs, Ipswich, MA, USA), 1 μL of primer, 1 μL of the probe, 4 μL of DNAse-free water, and 2.5 μL of DNA. A CFX Connect Real-Time System (BIO-RAD, Hercules, CA, USA) was used for thermal cycling using the following measures: initial denaturation at 95 °C for 1 min, followed by 60 cycles of denaturation at 95 °C for 15 s, annealing stage at 55 °C for 15 s, and finally elongation at 60 °C for 15 s. The quantitative PCR data were analysed using CFX Maestro Software 2.2 (Version 5.2, BIO-RAD).

### 2.4. Statistical Analysis

Statistical tests and related graphs were carried out using IBM SPSS Statistics (Version 28, IBM Corporation, Armonk, NY, USA) and GraphPad Prism (Version 9.7, San Diego, CA, USA). The chi-square test of independence was used to assess the associations between the categorical variables where applicable. The non-parametric Mann–Whitney U test was used to assess the associations between the study variables and the absolute copy numbers of cf-nDNA and cf-mtDNA. The level of significance was defined as *p* < 0.05. 

The cut-off values used to classify patients according to their levels of cf-nDNA and cf-mtDNA into “high” and “low” were calculated from the coordinates of the receiver operating characteristic (ROC) curve using Youden’s index. The Kaplan–Meier estimator was conducted to assess patient survival, stratified as “low” and “high” according to the copy numbers of cf-nDNA and cf-mtDNA. A Cox proportional hazards regression analysis was performed to estimate the univariate and multivariate hazard ratios of the study variables to predict the overall survival of the patients.

## 3. Results

### 3.1. General Characteristics of the Study Subjects

A total of 102 patients diagnosed with HNSCC were initially reviewed. Of these, 94 patients had follow-up data and considered eligible to participate (60 with laryngeal SCC and 34 with oral SCC). The mean follow-up time for all participants was 32.04 months (SD ± 19.1). The vast majority of the participants were males, accounting for 79 of the patients (84%), as shown in Table 2. The mean age of the study subjects was 61.5 years (SD ± 10.6) and ranged from 32 to 85 years, while the females were statistically significantly older than the males, at 69.9 years (SD ± 13.9) and 59.9 years (SD ± 9), respectively (*p* = 0.015). 

Most patients were smokers (75.5%), with a mean duration of smoking of 34 years (SD ± 11.9). Regarding the TNM stage of HNSCC at the time of diagnosis, more than half of the patients were diagnosed at stage IV (59.6%), followed by 25.5% of patients at stage III (Table 2). Fifty-four patients (57.4%) received chemo- and radio-therapy as part of their treatment plan, while 28 patients (29.8%) did not receive either chemotherapy or radiotherapy treatment.

At the end of the follow-up period, 43 patients (45.7%) were deceased, and the median survival time for the deceased patients was 12 months (range 1 to 38 months). The statistical analyses showed that the deceased events were not statistically significantly associated with gender, age, HNSCC site, TNM stages, smoking status and duration, or therapy (*p* > 0.05) (Table 2).

### 3.2. Correlations between Salivary cfDNA and cf-mtDNA with Subject Variables

**Figure 1:**
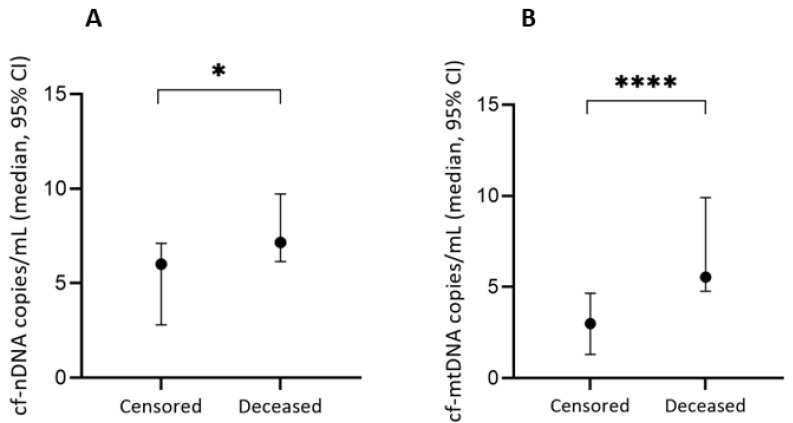
The association between the median copy number of saliva-based (**A**) cf-nDNA and (**B**) cf-mtDNA with the status of the patients (censored vs. deceased). Data are shown as mega copies/mL. * *p* < 0.05, **** *p* < 0.0005.

The absolute numbers of cf-nDNA and cf-mtDNA copies/mL were statistically significantly higher among deceased patients than among censored ones according to the Mann–Whitney U tests (*U* = 987.5, *z* = −1.988, *p* = 0.047 and *U* = 658.0, *z* = −4.209, *p* < 0.0005, respectively) (Table 2 and Figure 1). 

On the contrary, the absolute numbers of cf-nDNA and cf-mtDNA copies/mL were not statistically significantly associated with gender, age, smoking status and duration, TNM stages, or therapy (*p* > 0.05). 

**Figure 2:**
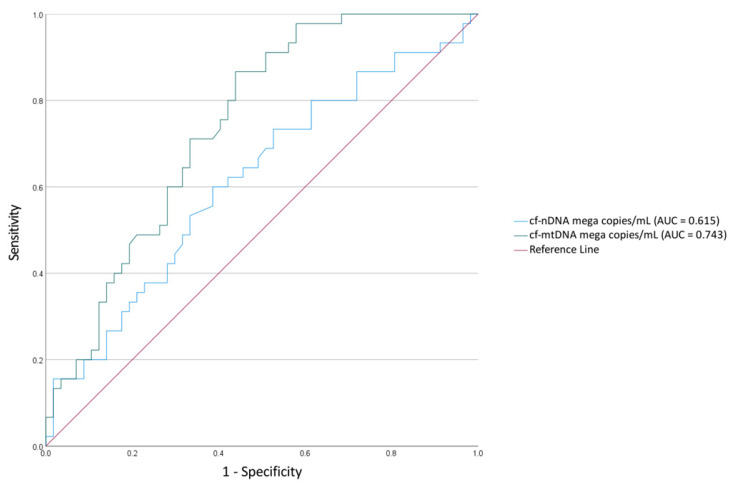
The receiver operating characteristic (ROC) curve used to determine the optimum cut-off points between “low” and “high” levels based on the cf-nDNA and cf-mtDNA yields of saliva samples according to the patient status (censored vs. deceased).

ROC curve analyses were performed to identify the optimal cut-off values to discriminate between deceased and censored individuals. Accordingly, the AUC was 0.615 (95% CI, 0.5–0.73) for cfDNA and 0.743 (95% CI, 0.65–0.84) for cf-mtDNA (Figure 2). Calculating the coordinates of the ROC curve showed that the thresholds above 7.0281 mega copies/mL for cfDNA and 3.0858 mega copies/mL for cf-mtDNA were elevated.

### 3.3. Prognostic Significance of Salivary-Based cf-nDNA and cf-mtDNA in Patients with HNSCC

**Figure 3:**
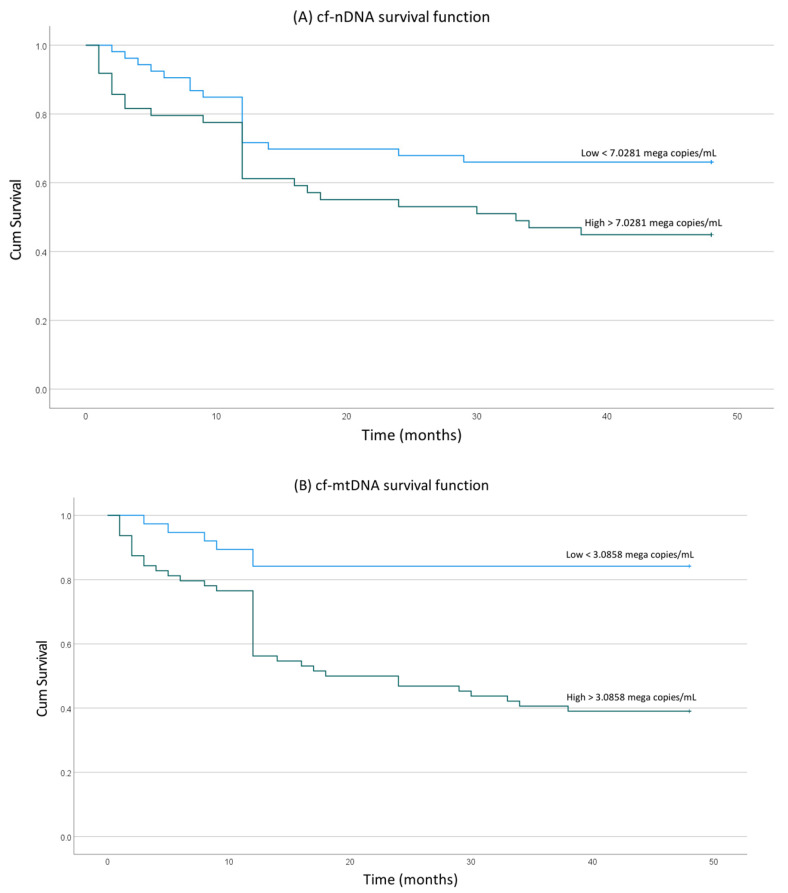
Kaplan–Meier estimates of the probability of overall survival of patients with HNSCC. Comparison between patients with higher and lower levels of (**A**) cf-nDNA and (**B**) cf-mtDNA.

The stratification of the patients according to their levels of cf-nDNA and cf-mtDNA into low and high based on the proposed thresholds was statistically significantly associated with a poorer overall survival. The cases with elevated levels of cf-nDNA had a statistically significant lower overall survival time of 28.4 months (95% CI, 22.9–33.8 months) than those with low cf-nDNA levels (overall survival time = 35.4 months, 95% CI, 30.6–40.2) (*p* = 0.035) (Figure 3A). Likewise, the patients with elevated levels of cf-mtDNA had a significantly lower overall survival time, equal to 26.3 months (95% CI, 21.6–30.9 months) than patients with low cf-mtDNA levels (overall survival time = 41.7 months, 95% CI, 37.1–46.3 months) (*p* < 0.0005) (Figure 3B).

Table 3 shows the results of the Cox proportional hazards regression models used to predict the hazard ratios for overall survival. Out of 10 variables, only the absolute numbers of cf-mtDNA copies/mL were statistically significantly associated with the patient’s outcome according to the univariate analysis (*p* = 0.009, HR = 1.01, 95% CI 1.0–1.02). However, introducing the multivariate analysis showed that three variables added significant values to the patient’s survival prediction: (i) the absolute numbers of cf-nDNA copies/mL (*p* = 0.013, HR 1.02, 95% CI 1.0–1.04), (ii) the absolute numbers of cf-mtDNA copies/mL (*p* = 0.002, HR = 1.01, 95% CI 1.0–1.02), and (iii) the TNM stage of HNSCC at the time of diagnosis (*p* = 0.045, HR = 0.44, 95% CI 0.19–0.98) (Table 3).

## 4. Discussion

Late diagnoses and poor prognoses are usually referred to when describing HNSCC in the literature. A negligible improvement was achieved in this area regardless of global efforts that have been carried out for decades [28]. To some extent, the current methods of managing HNSCC are primarily based on interpreting subjective clinical and pathological variables [6,19]. Therefore, it was not surprising to find that the majority of the patients with HNSCC in this study were diagnosed at late stages (stages III and IV), as this follows the global trend of the diagnosis of HNSCC [29]. Most importantly, this clearly shows the unfavourable impact of the interpretation inconsistency of HNSCC factors on patient management. Thus, the principal aim of this research was to employ molecular pathology for HNSCC screening and surveillance using well-established biomarkers. Our recent findings reported a significant increase in the concentrations of cf-nDNA and cf-mtDNA among patients with HNSCC, highlighting the potential objective applications of these molecules for surveillance purposes [19].

The head and neck region comprises various anatomical sites with diverse genetic and clinical presentations. This diversity has also been reported between adjacent sites within the oral cavity [6,8]. A previous study found that the expression of the X-linked inhibitor of apoptosis (XIAP) is significantly higher in SCCs on the floor of the mouth than in SCCs of the tongue [10], which was further associated with a significant decrease in overall survival [10]. Likewise, a gene profiling study revealed site-specific gene expressions in the head and neck region related to the aggressiveness of HNSCC [9]. This justifies the failure pattern of applying the prognostic factors across different HNSCC subsites [8]. These findings also support our results, where no significant association was found between the TNM stage of HNSCC and the deceased event. Likewise, our univariate progression survival analysis did not show a significant value for the TNM stage predicting the patient’s survival.

The findings of this study show that patients with high cf-nDNA levels were statistically significantly associated with a lower overall survival time. This finding is consistent with previous studies that found similar trends for cf-nDNA in oral cancers and other solid tumours [20,21,30]. Nonetheless, our progression survival analysis did not show a significant value for cf-nDNA as an independent predictor. Notably, the literature includes controversy regarding the tumour-specific prognostic value of cf-nDNA [31]. This can be attributed to cf-nDNA not being specific to malignant conditions, as it may also be influenced by various pathological and physiological conditions [17]. Thus, it has been proposed that cf-nDNA in HNSCC may be linked to tumour burden and comorbidities [30]. In support of this, a previous 15-year longitudinal study reported cf-nDNA as a predictor of all-cause mortality independent from other factors [17].

Unlike nuclear DNA, mitochondrial DNA is more susceptible to mutation and oxidative damage, making mitochondrial dysfunction a marked player during tumorigenesis [22]. The increased level of cf-mDNA is believed to be a part of a compensating mechanism due to the energy shortage in growing tumour cells [32]. Nonetheless, the literature includes very limited and sometimes contradictory data about the potential prognostic applications of cf-mtDNA in human malignancies [23]. Our results reveal that cf-mtDNA is associated with poor survival among patients with HNSCC, regardless of the HNSCC site (either oral or laryngeal), and that it is a significant predictor of the patient’s overall survival, independent from other variables. In line with this finding in the head and neck region, a previous project has reported the increased content of mitochondrial DNA as an independent predictor of overall survival in patients with oesophageal SCC using formalin-fixed paraffin-embedded (FFPE) samples [33]. 

On the contrary, there was no association between the mitochondrial DNA content in the FFPE samples of laryngeal SCCs and the overall survival of patients [34]. Notably, differences in the patterns of mitochondrial DNA have been reported not only between different malignancies but also among individual cancers [35]. Whilst the causal mechanism that explains these differences is not clear, they can be attributed to (i) the site of the mutation of the mitochondrial DNA D-loop, which is the region that mediates the mitochondrial DNA replication, or (ii) the dysfunction of the p53 gene, which is the gene responsible for maintaining mitochondrial genetic stability [36,37]. However, further research in this area while employing advanced molecular assessment tools is warranted to understand the influence of the HNSCC on cf-mtDNA and to explicate underlying molecular interactions. 

To the best of our knowledge, no previous study has assessed the prognostic utility of liquid biopsies in assessing cf-mtDNA levels, either in blood or in saliva. Therefore, this study provides a novel contribution to this field by providing an objective and non-invasive tool to monitor patients with HNSCC. Unlike other minimally invasive tools, which require a standard level of training [38], collecting saliva samples can be conducted at home by patients following simplified instructions. Considering the similar DNA genotyping profile between blood and saliva [39], further optimisations of this approach are required to adopt saliva-based liquid biopsies in the routine care of head and neck lesions.

This study has three potential limitations. Firstly, the number of included patients could have been higher based on a single centre, which may underestimate the prediction significance of some variables. Moreover, this also limits assessing the feasibility of this approach based on individual oral cavity subsites. Second, samples were taken once from each patient before therapeutic intervention. Therefore, it was impossible to assess the potential changes in cf-nDNA and cf-mtDNA levels through different stages of treatment. Finally, this study was only based on calculating the absolute number of the molecules of interest using qPCR. Therefore, further studies using next-generation sequencing are required to understand the underlying genetic interactions that may influence the levels of cf-nDNA and cf-mtDNA.

In conclusion, the saliva-based copy number of cf-mtDNA can be used as an independent predictor of the overall survival of patients with HNSCC. While this approach has the advantage of being objective and non-invasive, future studies using multi-centre large cohorts while employing a range of molecular assays are required to provide conclusive outcomes.

## Figures and Tables

**Table 1:** Probes and primer sequences of multiplex quantitative PCR assays.

Gene	Probes and Sequences of Primers (5′ → 3′)	Amplicon Lengths (bp)
Human beta-2-microglobulin (M17987)	Forward: GCT GGG TAG CTC TAA ACA ATG TAT TCA	94
	Reverse: CCA TGT ACT AAC AAA TGT CTA AAA TGGT	
	Probe: VIC-CAGCAGCCTATTCTGC	
Human mitochondrial (NC-012920)	Forward: CTT CTG GCC ACA GCA CTT AAA C	65
	Reverse: GCT GGT GTT AGG GTT CTT TGTTT	
	Probe: FAM-ATCTCTGCCAAACCCC	

**Table 2:** Main characteristics of the study variables at the end of the follow-up time and associations with patient status (deceased and censored).

Variables	Deceased n = 43 (45.7%)	Censored n = 51 (53.3%)	Total n = 94 (100%)	*p-*Value
Gender				
Male, *n (%)*	34 (79.1%)	45 (88.2%)	79 (84%)	0.227
Female, *n (%)*	9 (20.9%)	6 (11.8%)	15 (16%)	
Mean age; years (SD)	60.9 (10.9)	61.1 (10.1)	61.5 (10.6)	0.928
Smoking status				
Smokers, *n (%)*	33 (76.7%)	38 (74.5%)	71 (75.5%)	0.802
Non-smokers, *n (%)*	10 (23.3%)	13 (25.5%)	23 (24.5%)	
Mean duration of smoking; years (SD)	35.1 (11.8)	33.2 (12.2)	34 (12)	0.489
HNSCC site				
Oral, *n (%)*	19 (44.2%)	15 (29.4%)	34 (36.2%)	0.138
Laryngeal, *n (%)*	24 (55.8%)	36 (70.6%)	60 (63.8%)	
TNM stage				
Stage I, *n (%)*	4 (9.3%)	8 (15.7%)	12 (12.8%)	0.304
Stage II, *n (%)*	1 (2.3%)	1 (2%)	2 (2.1%)	
Stage III, *n (%)*	8 (18.6%)	16 (31.4%)	24 (25.5%)	
Stage IV, *n (%)*	30 (69.8%)	26 (51%)	56 (59.6%)	
Therapy				
Chemotherapy only, *n (%)*	5 (11.6%)	3 (5.9%)	8 (8.5%)	0.568
Radiotherapy only, *n (%)*	1 (2.3%)	3 (5.9%)	4 (4.3%)	
Both *, *n (%)*	23 (53.5%)	31 (60.6%)	54 (57.4%)	
None, *n (%)*	14 (32.6%)	14 (27.5%)	28 (29.8%)	
Saliva-based cf-nDNA median mega copies/mL	7.2	6.01	6.8	0.047
Saliva-based cf-mtDNA median mega copies/mL	5.5	2.9	4.7	<0.0005

SD: standard deviation, * both chemotherapy and radiotherapy.

**Table 3:** Cox proportional hazards regression analysis based on several prognostic variables, showing *p*-values, hazard ratios, and 95% confidence intervals.

Variables	Hazard Ratio	95% CI of Hazard Ratio	*p-*Value
Univariate analysis			
cfDNA copies/mL *	1.02	1.0–1.03	0.054
cf-mtDNA copies/mL *	1.01	1.0–1.02	0.009
Age *	0.99	0.97–1.03	0.76
Gender ^	0.68	0.34–1.37	0.276
HNSCC site ^	1.54	0.85–2.79	0.153
TNM stage ^	0.63	0.25–1.59	0.328
Smoking status ^	1.24	0.56–2.27	0.744
Duration of smoking *	1.0	0.98–1.13	0.732
Chemotherapy ^	1.17	0.63–2.17	0.632
Radiotherapy ^	1.68	0.93–3.02	0.086
Multivariate analysis			
cfDNA copies/mL *	1.02	1.0–1.04	0.013
Cf-mtDNA copies/mL *	1.01	1.0–1.02	0.002
Age *	0.99	0.95–1.03	0.691
Gender ^	0.66	0.29–1.49	0.321
HNSCC site ^	0.52	0.26–1.03	0.059
TNM stage ^	0.44	0.19–0.98	0.045
Smoking status ^	0.64	0.11–3.59	0.612
Duration of smoking *	1.01	0.97–1.05	0.6
Chemotherapy ^	1.71	0.69–4.22	0.243
Radiotherapy ^	0.39	0.17–0.93	0.063

* Continuous variable; ^ categorical variable gender, male vs. female; HNSCC site, oral vs. laryngeal; TNM stage, stage IV vs. other stages; smoking status, smokers vs. non-smokers; chemotherapy, received vs. did not receive; radiotherapy, received vs. did not receive.

## Data Availability

The data supporting this study’s findings are available on request from the corresponding author. The data are not publicly available due to privacy or ethical restrictions.
